# Linker histone defines structure and self-association behaviour of the 177 bp human chromatosome

**DOI:** 10.1038/s41598-020-79654-8

**Published:** 2021-01-11

**Authors:** Sai Wang, Vinod K. Vogirala, Aghil Soman, Nikolay V. Berezhnoy, Zhehui Barry Liu, Andrew S. W. Wong, Nikolay Korolev, Chun-Jen Su, Sara Sandin, Lars Nordenskiöld

**Affiliations:** 1 School of Biological Sciences, Nanyang Technological University, 60 Nanyang Dr., Singapore, 637551 Singapore; 2 NTU Institute of Structural Biology, Nanyang Technological University, Singapore, 639798 Singapore; 3 National Synchrotron Radiation Research Center, Hsinchu Science Park, Hsinchu, 30076 Taiwan; 4 Present Address: Department of Emerging Infectious Diseases, Duke-NUS Medical School, National University of Singapore, 8 College Road, Singapore, 169857 Singapore; 5 Present Address: Electron Bio-Imaging Centre (eBIC), Diamond Light Source, Harwell Science and Innovation Campus, Didcot, Oxfordshire UK; 6 Present Address: Singapore Center for Environmental Life Sciences Engineering, Nanyang Technological University, 60 Nanyang Drive, Singapore, 637551 Singapore

**Keywords:** Structural biology, Chromatin structure, Nucleosomes

## Abstract

Linker histones play essential roles in the regulation and maintenance of the dynamic chromatin structure of higher eukaryotes. The influence of human histone H1.0 on the nucleosome structure and biophysical properties of the resulting chromatosome were investigated and compared with the 177-bp nucleosome using Cryo-EM and SAXS. The 4.5 Å Cryo-EM chromatosome structure showed that the linker histone binds at the nucleosome dyad interacting with both linker DNA arms but in a tilted manner leaning towards one of the linker sides. The chromatosome is laterally compacted and rigid in the dyad and linker DNA area, in comparison with the nucleosome where linker DNA region is more flexible and displays structural variability. In solution, the chromatosomes appear slightly larger than the nucleosomes, with the volume increase compared to the bound linker histone, according to solution SAXS measurements. SAXS X-ray diffraction characterisation of Mg-precipitated samples showed that the different shapes of the 177 chromatosome enabled the formation of a highly ordered lamello-columnar phase when precipitated by Mg^2+^, indicating the influence of linker histone on the nucleosome stacking. The biological significance of linker histone, therefore, may be affected by the change in the polyelectrolyte and DNA conformation properties of the chromatosomes, in comparison to nucleosomes.

## Introduction

In eukaryotic cells, chromatin is a macromolecular complex mainly composed of DNA and histones. Cellular activities involving chromatin, such as DNA replication, gene transcription, DNA damage repair, DNA and histone modifications, carried out by different protein and RNA molecules rely on the dynamics that ensures accessibility of chromatin.

**Figure 1:**
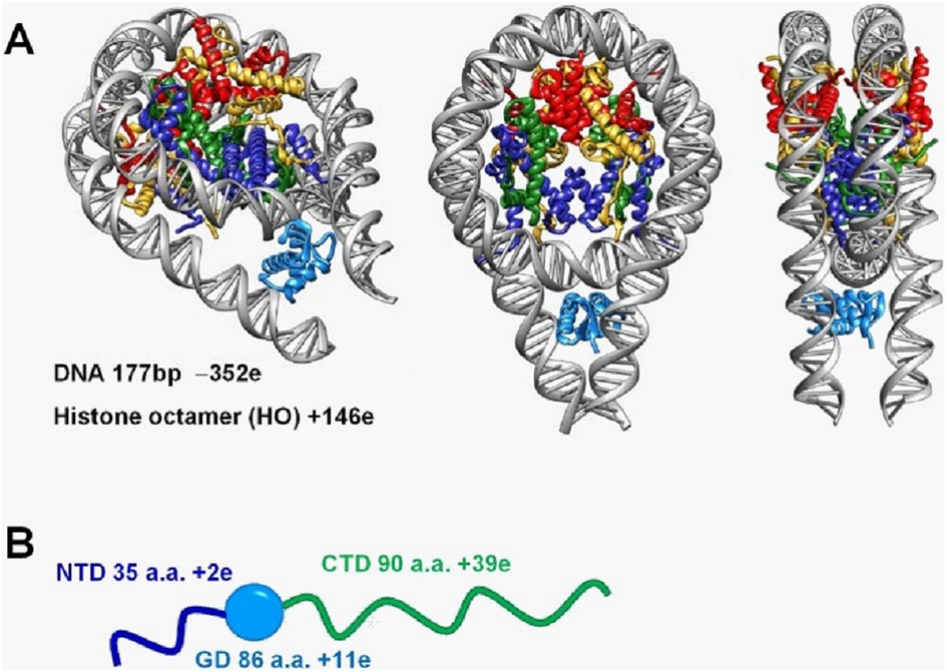
(**A**) 177 bp chromatosome, as a complex of the 177 bp nucleosome and linker histone H1. Three different views of the chromatosome are shown. The molecular structure was built using the crystal structure of the 197 bp chromatosome (5NL0.pdb^30^) by cutting 10 bp from each DNA end. A significant part of the unstructured histone tails and N-(NTD) and C-terminal (CTD) domains of the H1 are missing from the crystal structure. DNA is coloured grey, the globular domain of the H1 is light blue, core histones are coloured yellow (H2A), red (H2B) blue (H3), and green (H4). Structures were visualised using CHIMERA^83,84,94^ (www.rbvi.ucsf.edu/chimera/). (**B**). Schematic presentation of the H1 domain structure comprised by a central globular domain (light blue) and flexible N-(blue) and C-termini (green). Net charges of the chromatosome components and H1 domains are indicated. Microsoft PowerPoint 2016 (www.microsoft.com) was used to draw the image.

To fit into the limited space of the nucleus, DNA is packed by wrapping around a histone octamer (HO) by 1.7 turns, forming the basic unit of chromatin, the nucleosome core particle (NCP). The NCP consists of the HO, comprising of two copies each of the core histones H2A, H2B, H3 and H4, and 145–147 bp of nucleosomal DNA wrapped around the HO^1^. The highly basic N-termini of all eight histones protrude out of the core region of the HO. They are characterised by a disordered and flexible structure and can mediate nucleosome-nucleosome interactions. The tails are also accessible for various post-translational modification enzymes as well as surrounding DNA or proteins to modulate chromatin dynamics and gene regulation. Linker DNA of variable length (10–60 bp) connects nucleosomes into a 10 nm chromatin fiber^2^. At the next level of DNA packaging, linker H1 histones bind to the nucleosome at the dyad region interacting with linker DNA and the nucleosome forming the chromatosome^3^ (Fig. 1A), which leads to folding into so-called 30-nm chromatin fibers^4–6^ and higher-order structures in chromosomes. Although the existence of the 30-nm chromatin fibres in human mitotic chromosomes is disputed^7–9^, the 30 nm structure has been observed in vivo^10–12^.

The linker histone protein has a globular domain and two unstructured tails. The N-terminal domain (NTD) is short, and the C-terminal domain (CTD) is half the size of the whole molecule and is enriched in basic residues^13^ (Fig. 1B). The structure of the globular domain has been solved by X-ray crystallography to 2.5 Å resolution^14^. Still, the structural details of the interaction of the CTD and the NTD with the nucleosome and the linker DNA are not fully characterised. The highly positively charged CTD is suggested to be critical for the interaction of linker histone with nucleosome^15^. The linker histone binding to the NCP results in an increase of the DNA wrapping around the histone core from 1.7 to 1.9 turns and facilitates the formation of stem-like structures at the nucleosome entry-exit location^16^.

Numerous structural studies on isolated nucleosomes using linker histone variants from fruit fly, frog, chicken, mouse, and human have produced various structural models for linker histones binding (for reviews see e.g.^17–22^). The globular domain of the linker chicken histone H5 bound to a 167 bp nucleosome displayed a symmetric on dyad linker histone binding mode in the 3.5 Å X-ray structure^23^. In contrast, the H1 globular domain of the fruit fly was found to bind to the nucleosome asymmetrically in an off-dyad mode through two clusters of lysine residues located opposite to each other on the surface of the globular domain. One cluster binds to the nucleosomal DNA near the dyad, and the other interacts with 10 bp of one linker DNA. Two discrete lysine-rich short regions of the CTD are also involved in this chromatosome formation^24^. The Cryo-EM reconstructed structure of the 30 nm chromatin fibre also revealed an off-dyad localisation of the human linker histone H1.4 interacting with the nucleosome and both linker DNAs^6^.

The present structural knowledge shows a great variety of the linker histone—nucleosome interactions. The differences among various linker histone–nucleosome complexes seem to arise from: (1) Different linker histone variants/subtypes used resulting in diverse binding modes; (2) different types of nucleosomal DNA and varying lengths of the linker DNA; (3) different contexts: whether the interaction is between the linker histone is with a mono-nucleosome or within a nucleosome array. All these and possibly other factors could induce different binding modes.

A number of Cryo-EM studies, in combination with other techniques, substantially contributed to our knowledge about the structural variability of chromatin and its components^25–33^ (and references cited in recent reviews^26,33^). Multiple attempts have been undertaken to determine the structure of the chromatosome complex, and so far, the best description was obtained for a recent 197 bp structure^30^. The EM structure of this 197 bp (human) nucleosome with the *X. laevis* full-length linker histone H1.0, solved by single-particle analysis to 11 Å resolution revealed the on-dyad binding mode of H1. The globular domain interacts with the nucleosomal DNA at the dyad and the two linker DNAs in a lopsided fashion, appearing more intimately associated with one of the two linker DNA arms. The structure also suggested that the C-terminal domain binds to one of the linker DNAs, which endows the chromatosome complex with polarity.

To further advance the understanding of the linker histone—nucleosome interaction mechanisms, here we studied in vitro reconstituted nucleosomes and chromatosomes using a 177 bp DNA template containing the Widom nucleosome positioning sequence, human histone octamers, and the human linker histones H1.0. The molecular details of the interactions of the linker histone with the nucleosome were addressed using Cryo-EM single particle analysis. The properties of the nucleosome and the chromatosome in solution and in precipitated aggregates were investigated by using small-angle X-ray scattering (SAXS) in solution and with x-ray diffraction on Mg^2+^-precipitated samples.

## Results and discussion

### The Cryo-EM structure of the 177 bp nucleosome reveals a flexible linker DNA

The Cryo-EM single particle analysis method was used to reconstruct the structure of the 177 bp nucleosome and the 177 bp chromatosome as described in the Materials and Methods section and in Table 1. The nucleosome used in this study was reconstituted using the Widom 601 DNA sequence with 15 bp DNA extensions at both ends with bound linker histone to obtain a 177 bp chromatosome (details, see Materials and Methods). Furthermore, the human core and linker histone (H1.0) were used to understand the structure of a human chromatosome better.

**Table 1:** Cryo-EM data collection and image processing.

Sample	177 bp nucleosome	177 bp chromatosome
Molecular mass (kDa)	219	242
Sample conc. (mg/ml)	1.0	~ 0.58
Sample support	Quantifoil R2/2	Quantifoil R1.2/1.3
Microscope	Tecnai Arctica	Titan Krios
Detector	Falcon III	K2
Voltage (kV)	200	300
Pixel size/box size (Å)	1.28/216	1.1/264
No. movie frames	7	40
Mask diameter (Å)	210	180
Exposure time (s)	2.1	15
Electron dose (e^−^/Å^2^)	21	80
Micrographs recorded/used	1310/1304	766/564
Particles picked/used	81,608/60,086	348,898/46,196
Symmetry	C1	C1
Resolution (Å)	~ 12.5	~ 4.5

**Figure 2:**
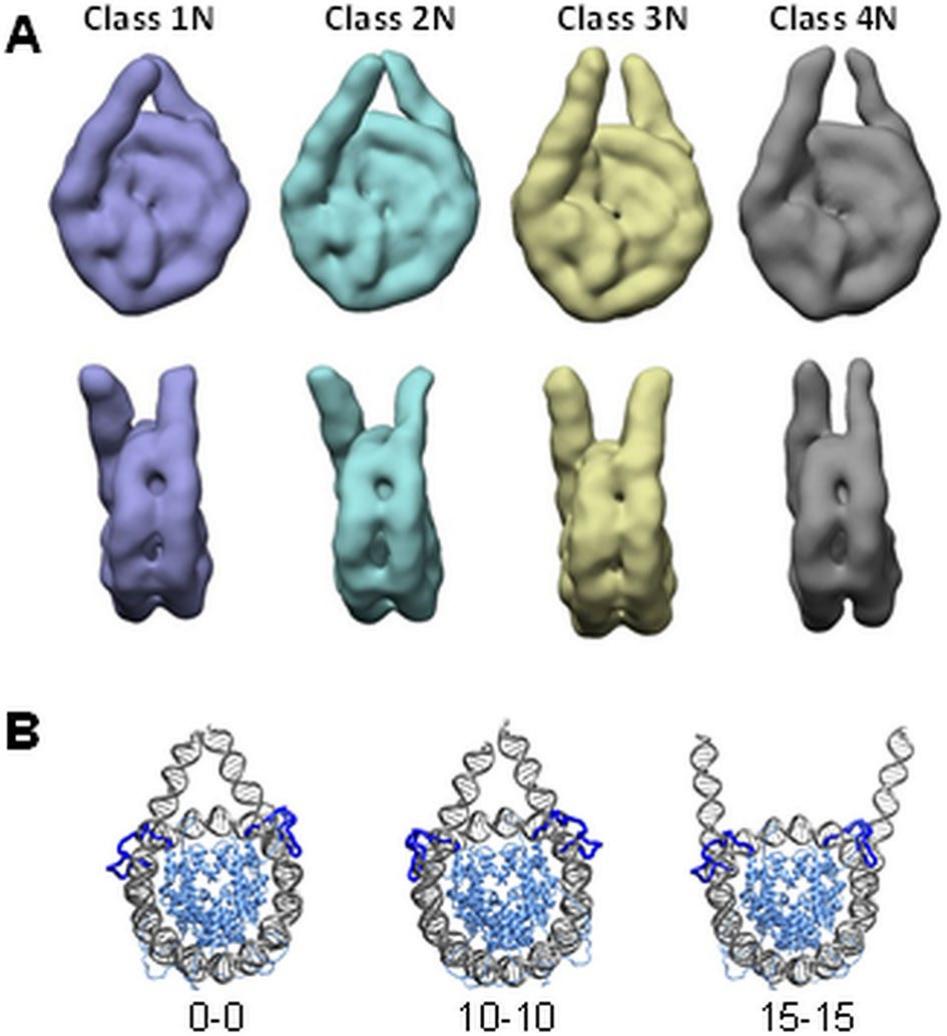
(**A**) 3D structure classification reveals diversified linker DNA geometry in the 177 bp nucleosome. Disk views of the four classes are displayed in the upper panel; side views are shown in the lower panel. (**B**). Molecular models of the 177 bp nucleosome, representing different degrees of DNA unwinding from the histone core. The numbers of the unwrapped DNA base pairs at the entry-exit sides are displayed below the corresponding structures. The long flexible N-termini of the two H3 histones that can extend and interact with linker DNA are highlighted in blue. The structure of the 147 bp NCP obtained from an MD simulation was used to build the snapshots. In the simulation, all histone tails collapse on the DNA as expected for the flexible oligocationic peptide tails at physiological salt conditions^95^. Physicochemical analysis^95^ and experimental data^96–98^ demonstrate that the H3 histone tails bind preferentially to the linker DNA, compared to the DNA of the NCP. The presence of the linker DNA allows tails extension and binding to this DNA. Structures were built and visualised using CHIMERA^83,84,94^ (www.rbvi.ucsf.edu/chimera/).

To discern how the binding of linker histone affects the structural and dynamic features of the 177 bp nucleosome, we first investigated the nucleosome in the H1 unbound state. The 177 bp nucleosome particles were classified into four classes based on diverse but limited orientations of the two linker DNA arms (Fig. 2A, classes are marked as 1–4 N; see also Fig. S2 showing images of the selected classes, and Table 1). The resolution of the 177 bp nucleosome density maps after refinement was estimated at 12 Å, following the gold standard (Fig. S2B). The comparison of the structures determined by Cryo-EM with molecular models (Fig. 2B) displaying different degrees of DNA unwrapping from the histone core suggests that DNA detachment from either side of the nucleosome likely does not exceed 10 base pairs. It may be noted that our modelling shows that the variation in the number of the unwrapped DNA base pairs in the range from 0 to 10 does not result in a noticeable change of the nucleosome appearance (compare 0-0 and 10-10 structures in Fig. 2B). However, a further increase of DNA unwrapping makes the nucleosome appear noticeably different (compare structures with 0-0 and 10-10 base pair unwinding to the one with 15-15 in Fig. 2B).

The structures of the 177 bp nucleosome display variable linker DNA conformations. Based on these Cryo-EM observations and results from solution SAXS studies (see below), we deduced that the two arms of the 15 bp linker DNA in the 177 bp nucleosome demonstrated pronounced flexibility in the absence of linker histone. The DNA is negatively charged, and one might expect that electrostatic repulsion between the linker arms may lead to the noticeable unwinding of the DNA from the histone core. This was indeed the case, but our data showed that the unwrapping did not exceed 10–15 bp on either side of the nucleosome. Larger unwinding is likely prevented by screening the DNA-DNA repulsion by the two long (45 aa) flexible positively charged (+ 14e each) N-termini of the H3 histones (highlighted in blue in Fig. 2B). These tails are located near the nucleosome dyad, can extend and interact and neutralise the linker DNA. Hence, charge neutralisation and screening by the H3 tails decrease the repulsion between linker DNA ends and reduce DNA detachment from the histone core. The flexibility of linker DNA is one of the essential factors defining interactions of chromatin with other protein factors in vivo, and this observation is therefore important within the context of the biological function of the nucleosome^27^.

### The 177 bp chromatosome displays an on-dyad H1 binding tilted towards one linker DNA arm

**Figure 3:**
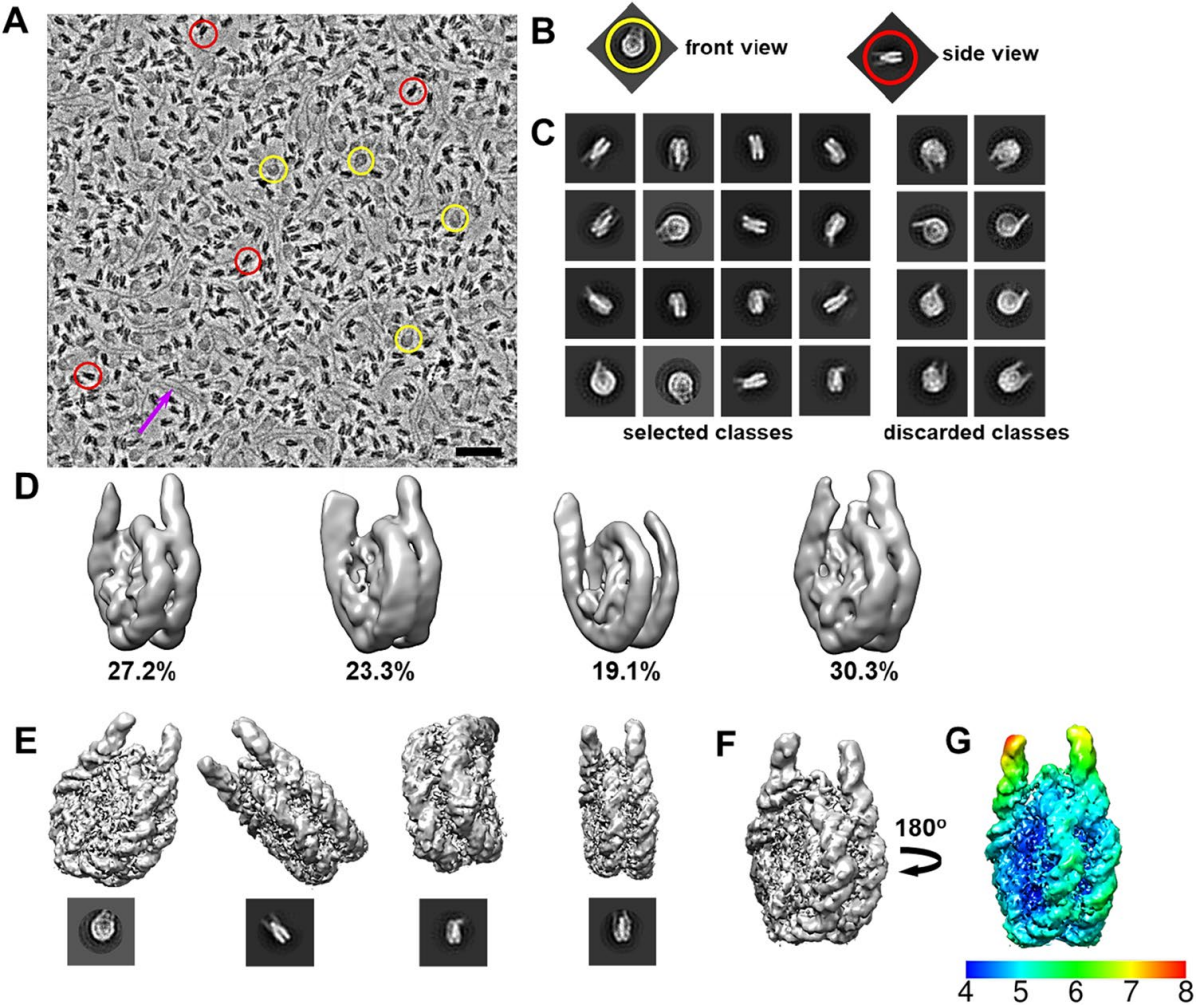
Cryo-EM processing for the 601–177 human chromatosome. (**A**) A representative electron micrograph of the 601–177 chromatosome recorded with the Volta phase plate at 500 nm defocus. The arrow points at free DNA. The scale bar is 40 nm. (**B**) Representative 2D class averages of the chromatosome generated in RELION 3.0 (**C**) Typical disk and side view 2D class averages. The corresponding particle views in the micrograph are circled. (**D**) Representative 3D class averages obtained from 168,042 particles selected from the 2D classification. (**E**) EM map of the chromatosome after 3D refinement of the right-hand 3D class comprising 46,196 particles. Different views are shown. The filtered local res map is shown at a contour level of 0.0205. The corresponding 2D class averages are placed below. (**F**) Cryo-EM map of the final 601–177 chromatosome after refinement. (**G**) Local resolution estimated in RELION^82,99^. The volumes maps were calculated by RELION^82,99^ (www3.mrc-lmb.cam.ac.uk/relion/index.php/Main_Page) and visualised using CHIMERA^83,84,94^ (www.rbvi.ucsf.edu/chimera/).

**Figure 4:**
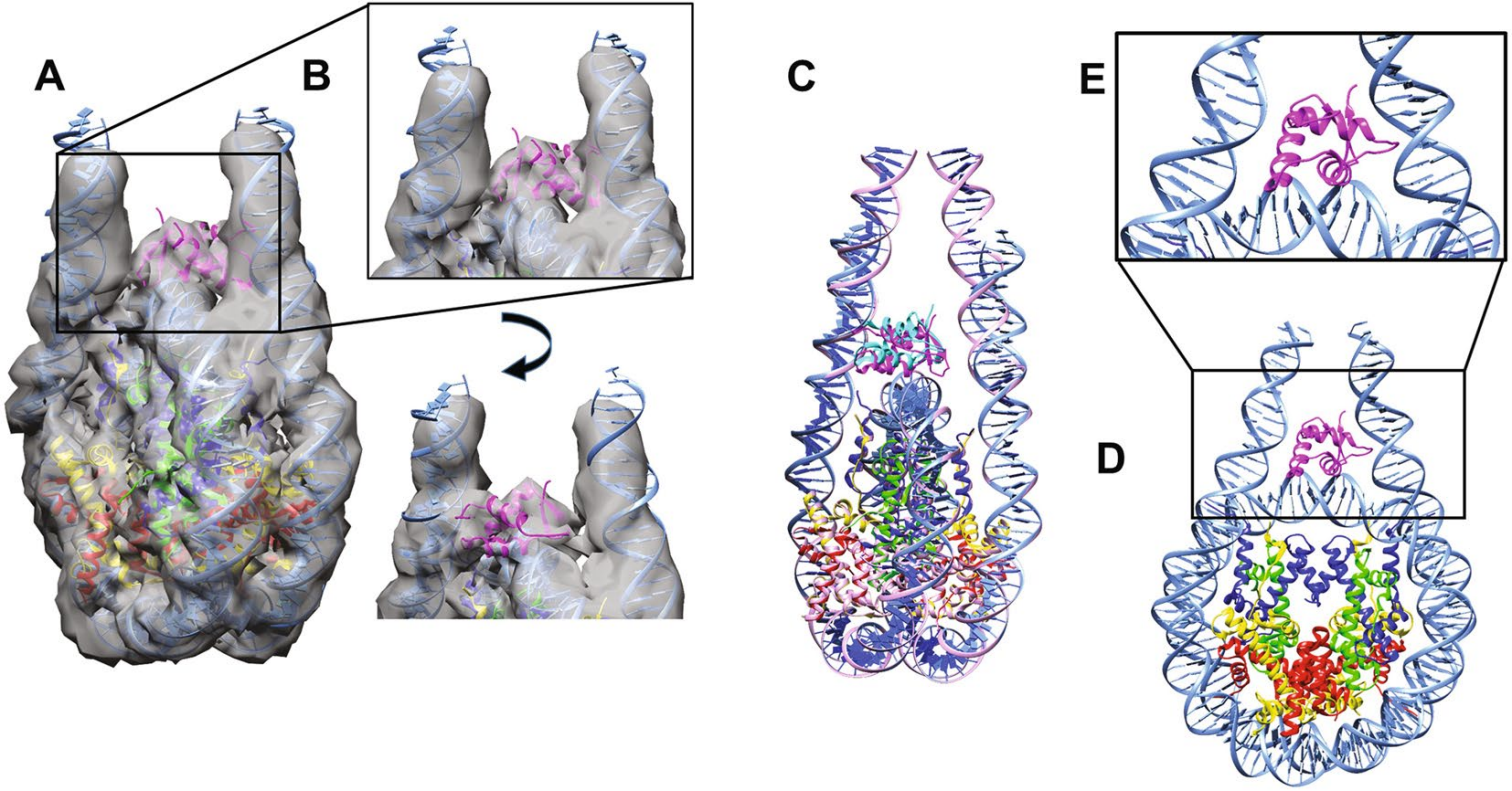
Cryo-EM structure of the 601–177 human chromatosome. (**A**) The model of the 601–177 chromatosome fits into the Cryo-EM map. The DNA (including linker DNA) is shown in blue. H2A is yellow, H2B is red, H3 is blue, H4 is green, and H1 is magenta. The filtered local resolution map is shown at a contour level of 0.0155. (**B**) Zoomed-in view of the region boxed in (A) showing three different views of the H1 bound to the nucleosome core particle, and fit of the H1 model into the EM map. (**C**) Comparison of the chromatosome (magenta) structure with the H1.5 bound 601–197 nucleosome in cyan (PDB entry 5NL0^30^). (**D**) Model of the 601–177 Chromatosome. (**E**) A zoomed-in view of the region boxed in (**D**). The winged helix fold (helix turn helix) of the linker histone H1 is shown in magenta. The DNA (including linker DNA) is drawn in blue. H2A is yellow, H2B is red, H3 is blue, and H4 is green. Structures were visualised using CHIMERA^83,84,94^ (www.rbvi.ucsf.edu/chimera/).

Next, we reconstructed the Cryo-EM structure of the 177 bp chromatosome, as described in the workflow schematically illustrated in Fig. 3. Data collection and image processing statistics are listed in Table 1. Figure 3A shows a representative defocused Volta phase plate (VPP) Cryo-EM image of the 177-chromatsome in vitreous ice. Excess free DNA can be seen in the micrograph, as highlighted by the magenta arrow. The different views of the typical 177-chromatosomes are distinguishable in the Volta phase plate image (Fig. 3A–C, black and white circles). Some representative 2D class sums are shown in Fig. 3B. Structural features such as core DNA, linker DNA, and core histones are easily recognisable in most 2d class sums, and in a few 2d class sums, an addition density at the linker region (indicated by the arrow) was also recognisable indicating the bound human H1.0 linker histone. The 3D classification showed less variability in the linker DNA orientation than in the 177 nucleosome structure (Fig. 2). The overall resolution of the density maps after 3D refinement (Fig. 3D–F) was estimated at 4.5 Å (Fig. S3). The significantly better resolution for the 177 chromatosome as compared to the 177 nucleosome is mainly due to the difference in microscopes and detectors used (see Materials and Methods). The local resolution map (Fig. 3G) shows that the linker histone region exhibits a significantly lower resolution compared to the core histones indicating variability/flexibility of the bound linker histone or even its lower occupancy. On the other hand, the resolution of the relatively more rigid regions of the core histones was sufficient to identify α-helices clearly. The core DNA was somewhat less resolved compared to the core histones indicating variations in the positioning of the DNA. The linker DNA beyond the region that was bound by H1.0 showed lower resolution implying a relatively flexible DNA even with the bound H1 protein. The electron density corresponding to the H1 linker histone was found to be centred at the dyad axis. We noted a strong density in the minor groove of DNA at the dyad indicative of H1 interaction with the DNA minor groove at dyad. Furthermore, the EM density map had a more pronounced density along one of the linker arms compared to the other arm (Fig. 3F), indicating an interaction of the linker histone in that region. The lower nucleosome and H1 resolution obtained here compared with recently published Cryo-EM structures of NCPs (without H1) at 3–4 Å resolution is not surprising. It is likely caused by the presence of the full-length C-terminal tail in the present construct that exhibits considerable dynamics, which leads to a more dynamic structure. The presence of the full-length C-terminal part of the linker histone affects the resolution^30^. The globular human hH1.0 X-ray structure (PDB ID: 6N89, chain B^34^) was used as a template to generate a putative homology model of the linker histone bound to the 177-chromatosome. The homology generated globular hH1.0 structure and the NCP-601L X-ray model (PDB ID: 3LZ0^35^) were fit into the EM density by visual inspection (Fig. 4A, B). (PDB ID: 3LZ0^35^). While the nucleosome core fits well, the globular H1 fit is far from perfect, with several clashes, which can be expected at the presented resolution. The imperfect fitting might be caused by several factors such as heterogeneity of the H1 binding contributing to this 3D class as well as the presence and variability in the dynamic association of the C-terminal tail with the linker DNA. This may result in the globular hH1.0 adjusting its structure upon binding at the dyad resulting in a deviation compared to the X-ray structure. The C-terminal domain is not part of the globular hH1.0 structure used in the fitting, while the experimental EM density that originates from the present chromatosome, comprises the full hH1.0, including the C-terminal tail. The additional density at the dyad axis between the linker DNA arms did fit reasonably well with the globular human hH1.0 structure (Fig. 4A, B). The rigid body fitting of the human H1.0 structure to the 177-chromatosome EM density suggests that the linker histone is centered on the dyad axis (Fig. 4D). Additionally, the extra density along one of the linker arms suggests that the H1 interacts more distinctly in that region (Fig. 4B). Therefore, this model is neither a strict symmetric nor an asymmetric model of linker histone binding to the nucleosome but reveals an on-dyad binding with H1 tilted towards one-linker DNA arm. Interestingly, this result is similar to that obtained by Bednar et al.^30^ in which they have proposed a similar structure for the 197 bp chromatosome reconstituted with *X. laevis* histones. They proposed an unevenly centred, so-called ‘lopsided’ model of the interaction of the linker histone with the nucleosome. When we overlapped the model of our 177 bp chromatosome with the 197 bp chromatosome (Fig. 4C), we observe that both the structures agree largely in terms of where the linker histone interacts with the nucleosome. There are differences between the placements of the linker histones in the EM densities between these two structures caused by the limited resolution of the maps. Based on these results we propose a structure of the human 177 bp chromatosome wherein the linker histone binds at the dyad axis of the nucleosome, in which it tends to interact with one of the linker arms (Fig. 4A) in a tilted manner. The proposed structure does not exclude a possible interaction with both the linker DNA arms, especially by the N-terminal and/or C-terminal tails of the linker histone. As noted above, compared to the 177 bp nucleosome, the chromatosome displays less flexible linker DNA arms. In addition, the unwinding of the DNA is less pronounced in the chromatosome due to the linker DNA association with the bound linker histone, in agreement with the observation made in the Cryo-EM study of the 197 bp chromatosome^30^.

### SAXS data reveals limited dynamic DNA end breathing of the chromatosome in solution

We wondered whether the structural behaviour of the 177 bp nucleosome and chromatosome confirms the features we observed with Cryo-EM. The effect of linker histone binding on the chromatosome structure in aqueous solution was therefore investigated using SAXS. The particle form factors define the intensity of the SAXS spectrum in isotropic solutions at low particle concentrations and at low salt^36,37^ (i.e., 10 mM KCl used in this work)^36,37^. Under such conditions, repulsive inter-particle interactions are weak, and there is a negligible contribution from the structure factor to the spectrum^36,37^. The shape and size parameters of the 177 bp nucleosome and chromatosome particles were obtained from the distance distribution analysis of spectra.

**Figure 5:**
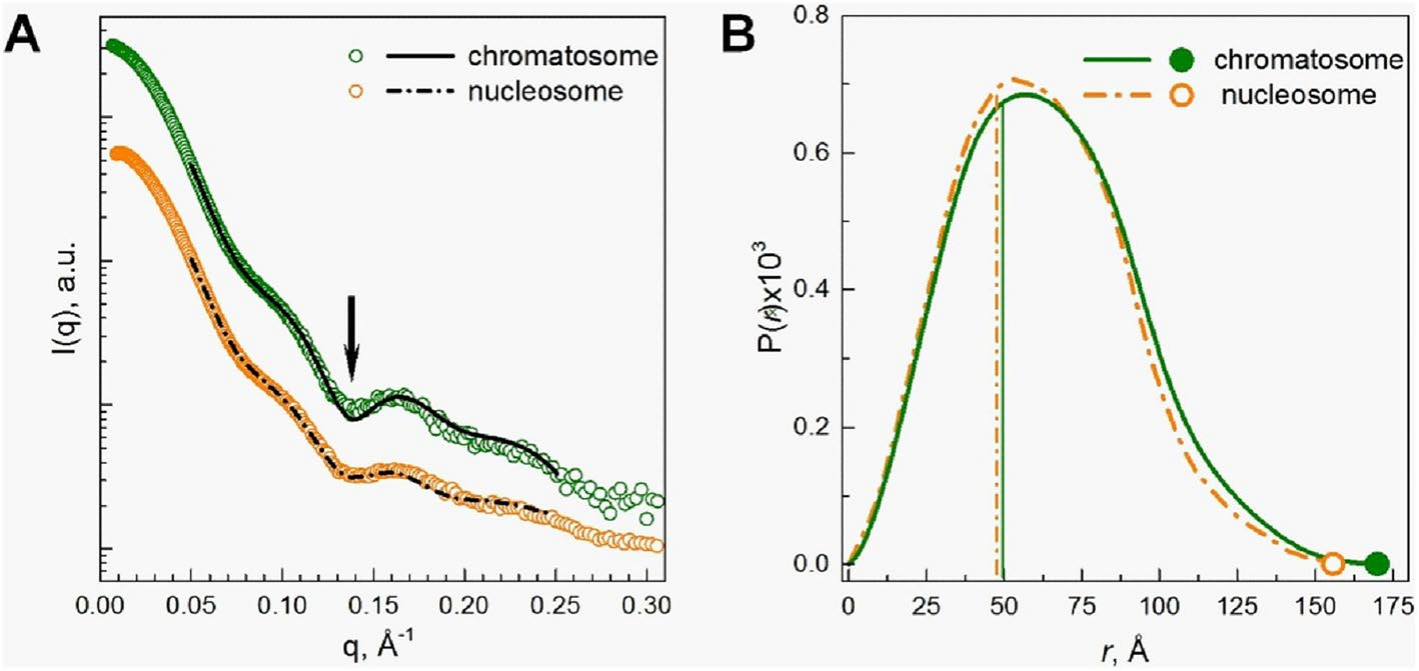
(**A**) Solution SAXS spectra, and (**B**) distance distribution functions *P(r)* calculated from the SAXS spectra of the 177 bp nucleosome (orange curve and points) and chromatosome (green curve and points). In (**A**), points are for the experimental data; black curves show the SAXS form factors calculated from molecular structures modelling the 177 bp nucleosome and chromatosome. For the chromatosome, the model was built using the crystal structure of the 197 bp chromatosome with linker DNA length adjusted (shown in Fig. 1); for the 177 bp nucleosome, the model was created using a snapshot of an MD simulation of the 147 bp NCP and stretches of straight linker DNA added. The arrow indicates the dip at q = 0.14 Å^−1^. In (**B**), vertical lines indicate values of R_g_; points are for D_max_ (maximal pair distance) determined for the nucleosome (orange) and chromatosome (green). See text for details and Fig. S3 and Table S4 of the Supporting Material for more fitting data. OriginPro software^100^ (www.originlab.com) was used to create the graph from the data generated by the ATSAS 2.8.4 package^91^ (www.embl-hamburg.de/biosaxs/software.html).

Molecular modelling of the particle structure allows the generation of simulated SAXS spectra that represent the form factor and that avoid the concentration and interparticle interaction effects that reduce the R_g_ and D_max_ values. Form factor spectra simulated using chromatosome and nucleosome models show good fits to the experimental SAXS profiles (Fig. 5A) with comparable R_g_ and D_max_ values (Table S2).

Based on the extracted radius of gyration, R_g_, and maximum distance, D_max_, values, the analysis of the experimental spectra shows that the chromatosome has an overall larger size than the nucleosome (Table S2). The difference in D_max_ is more significant in comparison with the difference in R_g_, indicating that the chromatosome size increase is accompanied by a change in shape, which makes the particle more asymmetric. It is in agreement with the Cryo-EM structure that displays a tilted on-dyad binding, which renders the shape some degree of asymmetry. In addition, it is likely that the presence of the long C-terminal tail of the linker histone as suggested by Bednar et al.^30^ is associated with one of the DNA arms, additionally contributing to the asymmetry of the shape of the chromatosome.

The solution SAXS results from both 177 bp nucleosome and chromatosome models with fully wrapped DNA or with the unwrapping of up to 10 bp were found to fit better to the experimental results, compared to models where unwrapping from either side exceeds 10 bp. This finding argues in favour of variable structures of the nucleosome and the chromatosome with dynamic but limited breathing of the DNA ends (Table S1), which is in agreement with the Cryo-EM observation of transient structures displaying linker DNA flexibility for the 177 bp nucleosome.

For a nucleosome core particle, a decrease of the depth of the depression at q = 0.14 Å^−1^ is a signature of the degree of the DNA unwrapping^37–39^. Figure 5A shows that the experimental chromatosome spectrum displays some difference from the spectrum generated from the crystal structure of the 197 bp chromatosome (with adjusted linker DNA length) in the region of q = 0.14 Å^−1^. The lower intensity in that area may be caused by more flexible linker DNA arms in solution compared to the crystal and Cryo-EM structures, which results in the transient unwrapping of DNA. DNA unwrapping from the HO was demonstrated to produce a comparable effect on the SAXS spectrum^40^ and observed in solutions of NCPs with tailless H3 and H4^39^. The increased values of R_g_ and D_max_ from the SAXS study suggest dynamic differences in the structure of the chromatosome compared to the nucleosome, and the result indicates an influence of the linker histone as a structural factor in linker DNA compaction of the nucleosome.

R_g_ values calculated from the distance distribution function P(r), from SAXS data for the 147 bp NCP, were previously reported to be around 42 Å in low salt conditions^36,40,41^. The R_g_ values of the 177 bp nucleosome (47.6 Å) and the chromatosome (49.4 Å) found in this work (Table S1) are more significant due to the presence of linker DNA, and linker histone, respectively. The increase of the R_g_ value of chromatosome relative to the nucleosome corresponds to about a 12% increase of the particle volume. This increase of the chromatosome volume roughly corresponds to the increase in size caused by the addition of the linker histone.

### Electrostatic interactions determine Mg^2+^-dependent self-association of the 177 bp nucleosome and chromatosome

**Figure 6:**
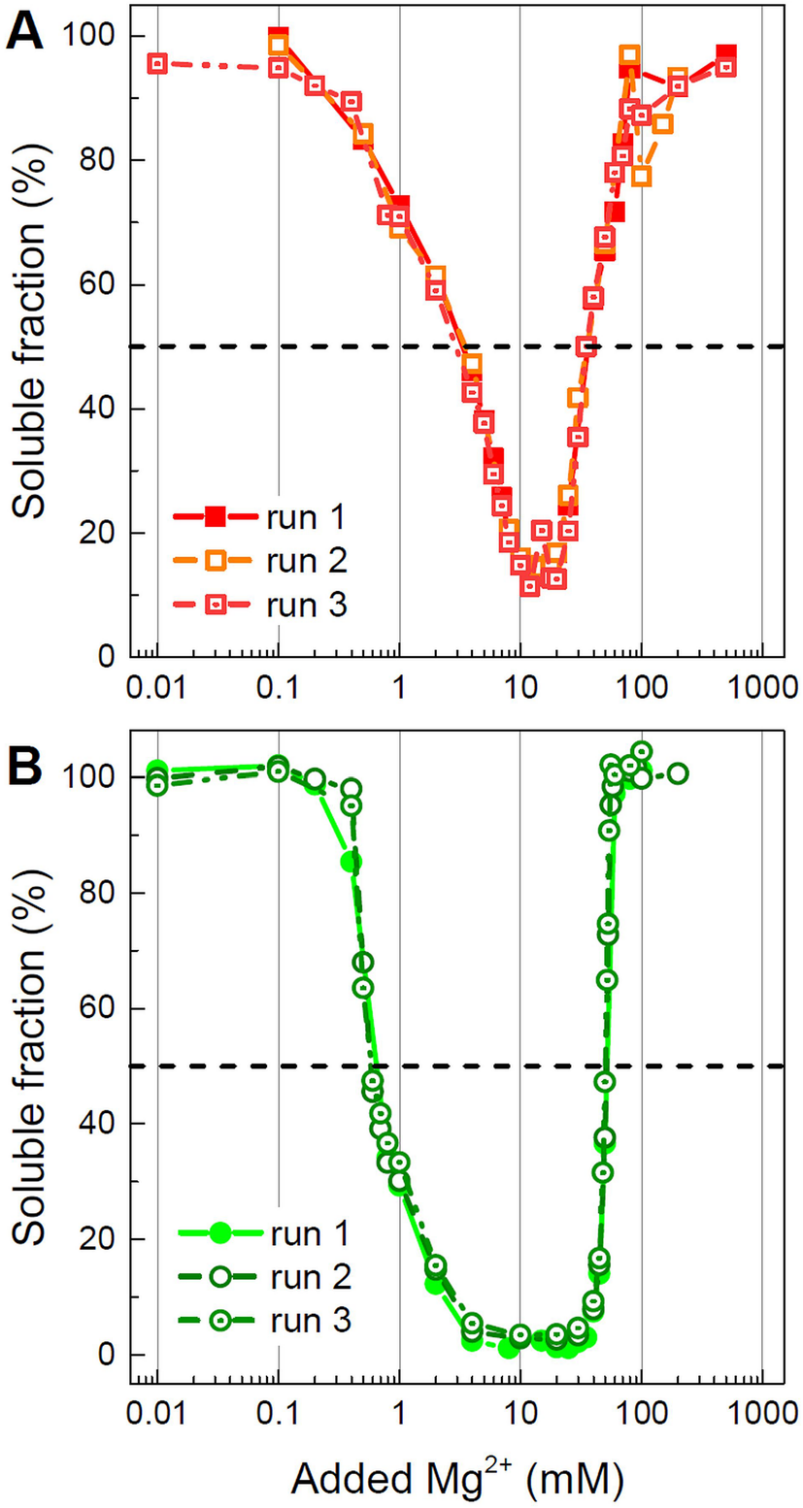
Mg^2+^-induced precipitation of the 177 bp nucleosome (**A**) and chromatosome (**B**) is reversible. The results of three independent measurements are shown for each chromatosome and nucleosome sample. In each graph, the dashed line shows 50% precipitation of the sample. OriginPro software^100^ (www.originlab.com) was used to build the graph.

Next, we investigated the influence of linker histone on nucleosome—nucleosome interactions by investigating the Mg^2+^-induced self-association in solutions of the 177 bp nucleosome and chromatosome. The addition of Mg^2+^ to the nucleosome or chromatosome solution screens the negative charge of the particles and changes the interaction between them from repulsive to attractive resulting in self-association (aggregation, often also referred to as “oligomerisation”)^42–57^. It results in a turbid solution that under the influence of gravitation leads due to a precipitated phase, which is in equilibrium with the supernatant containing the NCP (or chromatosome). Addition of Mg^2+^ leads to a gradual decrease in the amount of NCP in the solution until all the NCP has precipitated. Further increase of the Mg^2+^ may lead to resolubilisation of the precipitated phase^42,47,58–60^. In the precipitation assay (PA) experiments, the self-association was quantified by varying the Mg^2+^ concentration in the NCP/chromatosome solution in the range 0–500 mM, followed by centrifugation of the sample and measurement of the concentration of NCP/chromatosome in the supernatant. Subsequently, we determined the EC_50_ values from the PA curves (Fig. 6), defined as the Mg-concentration where 50% of the particles have precipitated out of solution due to the presence of the divalent cations (See Materials and Methods). Results of three different PA titrations were in good agreement (Figs. 6 and S5). Precipitation followed by resolubilisation at further increase of the Mg^2+^ concentration was observed for both the nucleosome and the chromatosome (Table S2). It may be noted that the supramolecular structure of the aggregated NCPs in the turbid solution or in the precipitated phase may be characterised with SAXS X-ray diffraction. It shows that the aggregates consist of domains of (hexagonal) columnar packed NCPs with a domain size of a few hundred nm, consisting of about 50 ordered NCPs (see below)^61^. These domains are the same whether detected by X-ray diffraction in the turbid solution or in the precipitate resulting from centrifugation of this solution (see below).

Cation induced self-association of the DNA, NCPs, and nucleosome arrays is governed by electrostatic interactions^62,63^ (and references cited therein) and determined by the net negative charge of these polyelectrolytes as well the cation valence and salt concentration. The 177 bp nucleosome carries an overall negative charge of − 206e comprised of the − 352e charge of the 177 bp DNA and + 146e net positive charge of the hHO. In the 177 bp chromatosome, the DNA negative charge is further neutralised to − 154e by binding one molecule of the linker histone (net charge + 52e). Correspondingly, the chromatosome requires less amount divalent cations present and exhibits aggregation at lower Mg^2+^ concentration, compared to the nucleosome, displaying a five times lower EC_50_ value (0.6 mM versus 3.0 mM, Table S2). This observation was also noted for the self-association of nucleosome arrays in the presence or absence of linker histone H5.

We also observed that Mg^2+^ induced nucleosome and chromatosome precipitation is reversible. At higher Mg^2+^ concentration, resolubilisation appears, and above 100 mM, the precipitation is reversed (Figs. 6 and S5). A similar phenomenon was also reported for solutions of NCPs^58^ and nucleosome arrays^42,47,59,60^. The data in Fig. 6 shows that both for the 177 bp nucleosome and the chromatosome, the precipitation transition proceeds in a broad range of Mg^2+^ concentrations (from 0.1 to 10 mM) compared to the reverse process of resolubilisation (between 10 and 100 mM). However, the resolubilisation of the 177 bp nucleosome begins right after the maximal precipitation at 10 mM Mg^2+^, while the chromatosome remains completely aggregated between 4 and 35 mM Mg^2+^ (Fig. 6). The EC_50_ resolubilisation values are equal to 34.2 and 51.2 mM Mg^2+^ for respectively the nucleosome and the chromatosome (Table S2).

The increased stability of the chromatosome condensed phase to resolubilisation might be due to the ability of the linker histone to crosslink chromatosomes, particularly by extension of long flexible, disordered, and positively charged CTDs. The linker histone is rich in positively charged lysine and arginine residues, and overall it has 52*e* net positive charge. The 177 bp chromatosome has 25% less negative charges compared with the 177 bp nucleosome. Binding of linker histone not only conferred electrostatic changes to the nucleosome but also strengthened the chromatosome self-association within the aggregates leading to a more stable state. This observation suggests possible biological significance in the control of chromatin accessibility by the linker histone-Mg^2+^ coordinated regulation. The divalent cation Mg^2+^ is a ubiquitous component of the cytoplasm and plays essential roles in genomic activities such as gene replication and transcription, in which cases chromatin de-compaction is required for the access of relevant enzymes or molecular machines. The different capability of Mg^2+^ in inducing nucleosome and chromatosome precipitation and resolubilisation might be important for regulating different states of chromatin compaction and de-compaction in vivo. It also indicates that the higher stability of the self-associated linker histone bound chromatosome may be a factor that stabilises the folded and aggregated chromatin in the presence of linker histones promoting heterochromatin in vivo.

### The Mg^2+^ precipitated chromatosomes form a lamello-columnar phase

Finally, we investigated the internal supramolecular structures formed in the Mg^2+^-induced self-associated 177 bp nucleosome and chromatosome precipitates using SAXS X-ray diffraction measurement. It was previously shown that NCPs form a columnar hexagonal phase made of domains (microcrystallites) with 20–30 columns, each consisting of 40–50 NCPs with a domain size of a few hundred nm^61^. It may also be noted that the hexagonal columnar phase can be detected either by direct SAXS experiments on the turbid solution or on the pellet obtained after centrifugation, resulting in identical ordered phase^40,61^.

**Figure 7:**
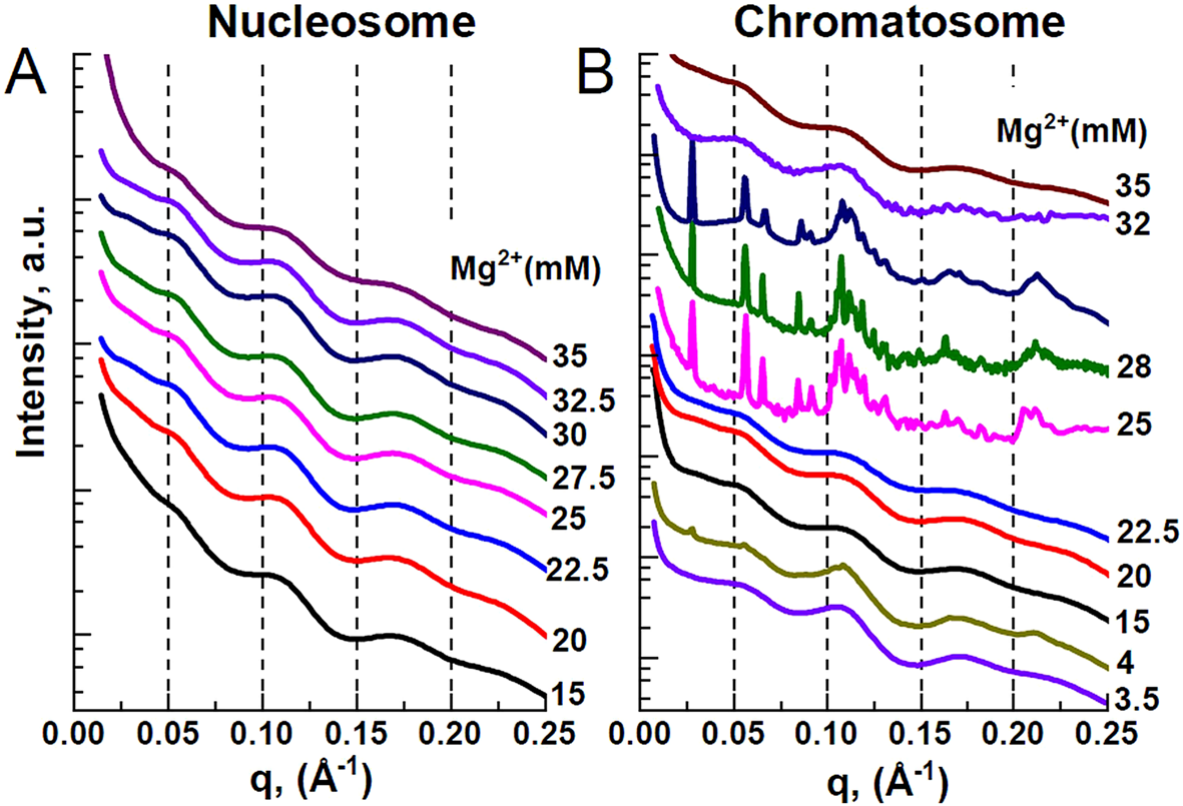
SAXS spectra of the nucleosome (**A**) and chromatosome (**B**) samples precipitated from 8 mg/mL solution by the addition of Mg^2+^. The concentration of Mg^2+^ is indicated next to the spectra. In (**B**), the most prominent peaks are at 0.0278, 0.056, 0.066, and 0.1075 Å^−1^ (see also Fig. S6). The peak at 0.1075 Å^−1^ and its second-order reflection at 0.21 Å^−1^, indicated as q_1h_ and q_2h_, respectively, originate from chromatosomes stacked into columns. OriginPro software^100^ (www.originlab.com) was used to create the graph.

**Figure 8:**
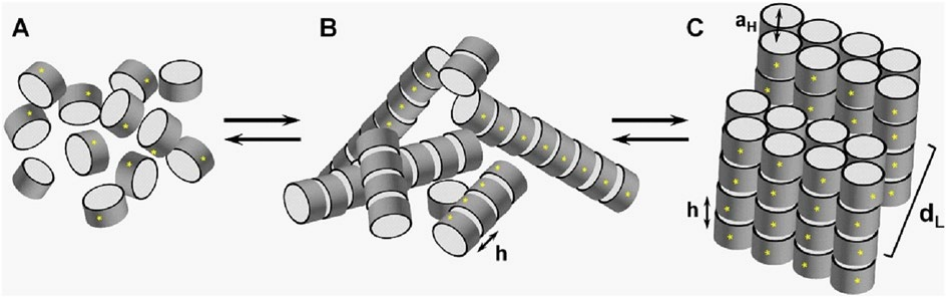
Schematic presentation of different nucleosome/chromatosome phases. (**A**). Isotropic solution with repulsive interaction between the particles. (**B**). Columnar phase without inter-columnar order. The stacking distance, h, characterises the separation between nucleosomes or chromatosomes within the column. (**C**). The lamella-columnar phase observed for the 177 bp chromatosome; a_H_ is an inter-columnar distance within a bilayer; d_L_ is the distance between bilayers. The yellow dots indicate the facing direction of the nucleosome dyad or location of linker DNA in the chromatosome. Adapted from^61^ with permission from Elsevier.

The resulting SAXS spectra are shown in Fig. 7. The supramolecular structural arrangements of the nucleosomes and chromatosomes in the aggregated phase that may be inferred from the X-ray diffraction data are displayed in Fig. 8B, C. Aggregated nucleosomes produced spectra with broad scattering peaks centred around 0.05, 0.11, and 0.175 Å^−1^ in the studied range of 15–35 mM Mg^2+^ (Fig. 7A). The most prominent peak around 0.11 Å^−1^ originates from the stacking of nucleosomes. The stacking via the nucleosome core region results in the formation of columns of chromatosomes. The local alignment of columns results in the 0.05, and 0.175 Å^−1^ peaks and contributes to the 0.11 Å^−1^ peak. The phase formed by nucleosomes is characterised by short-range interactions and long-range disorder, which can be termed columnar isotropic (Fig. 8B)^61^.

The aggregated chromatosomes were studied in comparable ranges of cation concentrations, and a different phase behaviour was identified. In the range of 25–30 mM Mg^2+^, the Bragg diffraction peaks are characteristic of the presence of a highly ordered phase formed by chromatosomes (Fig. 7B; see also Fig. S6). These peaks correspond to the separation of the chromatosomes inside the columns h = 58.45 Å. The peak q_1_ = 0.066 Å^−1^, and its second-order peak q_2_ = $\sqrt{3}$×q_1_ = 0.11 Å^−1^ originate from the alignment of chromatosome columns into an ordered hexagonal lattice with an inter-columnar distance at a_H_ = 110 Å. The peak q_1L_ = 0.0278 Å^−1^ and its second reflection q_2L_ = 2 × q_1L_ = 0.056 Å^−1^, corresponding to the distance of 226 Å are proposed to originate from the arrangement of the columnar hexagonal phase into bilayers of columns separated by an aqueous gap, termed the lamello-columnar phase^64^, shown in Fig. 8C. Outside of the 25–30 mM Mg^2+^ range, chromatosomes form a columnar isotropic phase (Fig. 8B), similar to nucleosomes (Fig. 8A, B).

The lack of long-range ordered structure in aggregates of 177 bp nucleosomes may reflect the flexibility of the two linker DNA arms, in agreement with the results of the Cryo-EM structure of the 177 bp nucleosome, which revealed variable conformations of the linker DNA. The formation of the bilayers is suggested to be due to the asymmetric shape of the 177 bp chromatosomes that prevents the formation of a continuous lattice of hexagonally ordered columns. Both the stacking distance (h = 58.5 Å) and inter-columnar distance (a_H_ = 110 Å) of the 177 bp chromatosome are comparable with those found in condensed phases of NCPs being a_H_ = 110–112 Å and h = 55–57 Å^61^. The stacking of nucleosome core particles and chromatosomes is mediated by core histone and histone tail interactions^56,65^. The comparable distances indicate that the binding of linker histone does not affect the columnar parameters outside the linker histone binding region. The 177 bp chromatosome lacks the symmetry of the 145–147 bp nucleosome core particles, and therefore the chromatosome forms bilayers of columns instead of the continuous hexagonal phase. The chromatosome appears to be flatter and more rigid, in comparison with the 177 bp nucleosome in the linker DNA-linker histone interaction area, due to the linker histone binding. The bilayers of chromatosomes may have their dyads ordered to face the solution (Fig. 8C). Linker histones may contribute to the formation of the bilayer phase by bridging the nucleosomes in the columns. Such ordering may result in additional correlations that would produce diffraction peaks in 2D spectra. The significant difference in the inter-bilayer distance between the 177 bp chromatosome (d_L_ = 226 Å) and the values of 358–376 Å^66^ reported for NCPs forming the lamello-columnar phase may be due to differences between well-defined recombinant particles aggregated by divalent cations in this work, and purified particles from a natural source of higher heterogeneity and aggregated by low monovalent salt and osmotic stress^64,66,67^.

## Conclusions

The dynamic structures of chromatin play essential roles in genomic activities within living cells. Chromatin fibres adopt different levels of compaction to cope with various cellular activities. Various factors contribute to the formation and maintenance of the diversity of chromatin structures. In this work, we studied a chromatosome formed from a 177 bp DNA sequence comprising of the Widom ‘601′ positioning sequence nucleosome, human HO and the human H1^0^ linker histone. Cryo-EM single particle analysis, SAXS, and precipitation assay methods were used to study the structure of these well-defined model systems.

In summary, the linker histone displays a tilted (‘lopsided’) binding at the dyad, and the interaction with linker DNA affects the structure and dynamical properties of the particle. Cryo-EM shows the binding position and the effect of lateral compaction of linker DNA arms. This affects the stacking properties of chromatosomes and allows them to form a lamello-columnar phase. Precipitation assay studies demonstrate the change in surface charge. The solution SAXS study shows that the overall shape of the chromatosome particle is not significantly affected by linker histone binding, although the chromatosome may display dynamic linker DNA arms with partial unwrapping from the HO core. In agreement with the Cryo-EM results, the SAXS measurements show that the nucleosome and the chromatosome have similar spherically averaged shapes and sizes, though linker DNA in the 177 bp nucleosome displays variable and partially unwrapped structural features. Precipitation assay and SAXS results show that the linker histone bound nucleosome is significantly more stable (over a larger Mg^2+^ concentration range) compared to the nucleosome itself, and forms a highly ordered phase, contrary to the nucleosome that lacks long-range ordered structure. These observations may be important within the context of linker histone bound chromatin, and these features may promote condensed heterochromatin in vivo. The findings of this study contribute to the understanding of the linker histone role in nucleosome structure and dynamics and contribute to the recent Cryo-EM^25,27–29^ and NMR advances^68–70^ in revealing the structural heterogeneity of chromatin and its components.

## Materials and methods

### Preparation of the 177 bp nucleosome and chromatosome

The construct of DNA containing 12 tandem 177 bp repeats of the 601 Widom nucleosome positioning sequence^71^ (12-177-601 DNA; a gift from Prof. Timothy J. Richmond ETH Zürich, Switzerland) was amplified and purified as described^59^. Briefly, the plasmid (pWM530 vector + 12-177-601 DNA) was transformed and amplified in Top10 *E. coli* cells grown at 37 °C with 225 rpm shaking for at least 18 h. Cells were collected by centrifugation at 7000 rpm for 7 min. The 12-177-601 plasmid was isolated by alkaline lysis followed by the RNase A treatment at 37 °C overnight, subsequent phenol extraction and ethanol precipitation. The 12-177-601 DNA was released from the pWM530 vector by EcoRV digestion at 37 °C overnight. Separation of 12-177-601 DNA was done by PEG fractionation followed by size exclusion chromatography. The single repeat of 177 bp 601 DNA was obtained from the ScaI digestion of the 12-177-601 DNA, followed by phenol extraction and ethanol precipitation. DNA, histone, nucleosome, and chromatosome concentrations were determined from UV-absorption (NanoDrop 2000/2000c spectrophotometer, Thermal Scientific) using known extinction coefficients.

Preparation of the *Homo sapiens* recombinant core histones H2A, H2B, H3 and H4 was carried out separately for each histone as described in^72^. The histone plasmid was transformed and expressed into *E. coli* BL21(DE3)pLysS competent cells. Cells were cultured at 37 °C with 225 rpm shaking. Histone expression was induced by 0.4 mM IPTG at OD_600_ = 0.6. Cells were harvested 3 h after the induction by centrifugation at 7000 rpm for 7 min. Cell pellets were re-suspended in wash buffer (50 mM Tris–HCl, 100 mM NaCl, 1 mM β-mercaptoethanol). Cells were broken down by sonication with 25% amplitude, 1 s on and 1 s off for 1 h or until homogeneous. The inclusion bodies containing the desired histone were harvested by centrifugation at 18,000*g*, 4 °C for 15 min. Pellets were washed three times in 150 mL washing buffer (first two times with 1% Triton X-100), followed by centrifugation at 18,000*g*, 4 °C for 10 min after each wash. The histones were purified by size exclusion chromatography using the Hiprep 26/60 Sephacryl S-200 h column, followed by cation exchange chromatography with the Resource S column.

Histone octamer refolding and purification were performed according to the published procedures^73^. Histones H2A, H2B, H3, and H4 were dissolved separately in the unfolding buffer (7 M guanidinium HCl, 20 mM sodium acetate, 10 mM DTT) to concentration 2–3 mg/ml. H2A, H2B, H3, and H4 were mixed in an equimolar ratio, and concentration was adjusted to 1–2 mg/ml by unfolding buffer. Refolding of the histone octamers was achieved by triple 5-h dialysis against 1L of refolding buffer (2 M NaCl, 10 mM Tris–HCl, 1 mM EDTA, 10 mM β-mercaptoethanol) at 4 °C. Aggregates were removed by centrifugation at 18,000*g* for 10 min. The supernatant with the histone octamers was concentrated by the Amicon Ultra centrifugal filter (50 KDa cutoff) with 3000*g* to a final concentration ~ 10 mg/ml. The HO was purified by size exclusion chromatography using the Hiload 16/600 Superdex 200 pg column. Fractions of the purified HO were pooled and concentrated by the Amicon Ultra filter to ~ 10 mg/ml and stored in 50% glycerol at − 20 °C.

Preparation of linker histone H1,0. The *H. sapiens* H1.0 histone plasmid DNA construct was a gift from Curt Davey (School of Biological Sciences, NTU, Singapore). The plasmid was transformed and expressed in *E. coli* BL21(DE3)pLysS competent cells. Cells were cultured at 37 °C with 225 rpm shaking. Histone expression was induced by 0.4 mM IPTG at OD_600_ = 0.6. Cells were harvested 3 h after the induction by centrifugation at 7000 rpm for 7 min. The linker histone purification protocol was adapted from^60^. Cell pellets were suspended in 50 mM Tris–HCl pH 8.8, 500 mM NaCl. Cell lysis was done by sonication with 1 s on and 1 s off at a 25% amplitude for a total of 1 h. The homogenised cell lysate was double centrifuged at 35,000*g*, 4 °C, for 30 min. The purification was done by two-step cation exchange chromatography with the SP Sepharose FF column, followed by the Resource S column. The pure linker histone was lyophilised and stored at − 80 °C.

In vitro reconstitution of nucleosomes was performed according to the published work^60,73,74^. Due to the uncertainties in measurements of the HO concentration, unescapable aggregations during salt dialysis, and other ill-defined reasons, to obtain saturated nucleosome samples, the hHO titration was carried. Titrations were performed in 50 µL scale in 2 M NaCl and 10 mM DTT with fixed DNA concentration (6 µM, 0.65 µg/µL), whereas the hHO amount was varied using different hHO to DNA molar ratios around 1:1. The nucleosome reconstitution was achieved by stepwise dialysis against buffers with lowering salt concentrations: 0.85 M (3 h), 0.5 M (3 h), 10 mM NaCl (twice, 5 h each time). Aggregates were removed by centrifugation (10,000 rpm × 5 min), and reconstitution quality was assessed by EMSA on 6% PAGE, with freshly made 0.25 × TBE running buffer, at 120 V for 50 min.

The in vitro reconstitution of chromatosome was divided into two parts: reconstitution of nucleosome and addition of linker histone. The nucleosome reconstitution mixture was prepared using hHO to DNA ratio determined as optimal in the reconstitution of pure nucleosome followed by dialysis against 0.85 M and 0.6 M NaCl buffer (20 mM HEPES pH 8.0, 1 mM DTT, 1 mM EDTA). The nucleosome sample was recovered at this point and used for titration by the H1.0 histone. A broad linker histone titration was first applied with H1.0 to nucleosome molar ratio ranging from 0.5:1 to 3:1 on a 50 µL scale starting from 0.6 M dialysis buffer. For proper binding of the linker histone to the nucleosome, the nucleosome—H1.0 histone mixture went through overnight dialysis at 4 °C against 10 mM NaCl buffer (20 mM HEPES pH 8.0, 1 mM DTT, 1 mM EDTA). Dialysis buttons were bagged in the 3000 MWCO membrane tubing containing 20–30 mL of 0.6 M buffer, which was then put in the 2L reservoir of the 10 mM buffer. Samples were centrifuged at 9000*g*, 4 °C for 5 min; the supernatant was analysed by EMSA on 5% PAGE. Based on the result of broad H1.0 titration, a narrow range of the nucleosome: H1.0 was set up for a preparative narrow linker histone titration.

### 177 bp nucleosome and 177 bp chromatosome Cryo-EM sample preparation and data processing

Approximately, 4–5 μl of either the 177-nucleosome sample at ~ 0.4 mg/ml or the 177-chromatosome sample at ~ 0.58 mg/ml in a buffer containing 20 mM Tris pH 7.5 and 1 mM DTT was applied to a freshly glow-discharged holey carbon grid (177-Nucleosome: Quantifoil R2/2 Cu 200 mesh, 177-Chromatosome: R1.2/1.3 Cu 200 mesh), blotted for 2 to 3 s, and plunged into liquid ethane using the Vitrobot (FEI, Thermo Scientific) plunge freezer.

The cryo-grids with 601–177 Nucleosome particles were imaged using the FEI Tecnai Arctica cryo transmission electron microscope, which was equipped with a 200 kV field emission gun, and Falcon III direct electron detection camera. Micrographs were collected at 78,000× nominal magnification, corresponding to an object pixel size of 1.28 Å in movie mode with 20–25 e^−^/Å^2^*s electron dose at various defocus values (− 1.5, − 1.75, − 2.0, − 2.25, − 2.5, − 2.75, − 3.0 µm). Movie frames were motion-corrected^75,76^, and the initial evaluation of the micrographs was done in EMAN2 to discard those with astigmatism or with very few or very crowded particles. The estimation of the CTF parameters was done by using the CTFFIND3 program^77^. Particles were picked manually in EMAN2 by e2boxer.py^78^. The rest of the image analysis was performed in RELION 2.0^66^. 2D and 3D classes and the 3D refined model of the 177-chromatosome can be seen in Fig. 3. The microscope settings, imaging conditions and image analysis statistics are mentioned in Table 1.

An initial dataset of the 177-chromatosome was also recorded on the Tecnai Arctica (FEI, ThermoFisher Scientific) at 200 kV equipped with a Falcon 3 camera to evaluate the quality of the sample. Micrographs were collected at 78,000× nominal magnification, corresponding to a pixel size of 1.28 Å in movie mode with a total dose of ~ 25 e-/Å2, recorded at various defocus values (− 1.5, − 1.75, − 2.0, − 2.25, − 2.5, − 2.75, − 3.0 µm) (Table S1). Subsequently, electron micrographs were recorded on Titan Krios (FEI, ThermoFisher Scientific) at 300 kV equipped with a Gatan GIF Quantum energy filter and a K2 Summit (Gatan, ThermoFisher Scientific) direct electron detector in counting mode. Data was collected in nanoprobe mode at parallel illumination with the Volta phase plate (FEI, ThermoFisher Scientific). A total dose of ~ 80 e/Å^2^ was fractioned into 40 frames. The microscope settings and imaging details are further listed in Table S1. Data was collected between 350 and 900 nm under-focus. Astigmatism and coma-free alignment were corrected using AutoCTF, a software tool by FEI (ThermoFisher scientific) for correcting astigmatism and coma-free alignment of the microscope.

Warp^79^ was used to perform on-the-fly processing of the electron micrographs to evaluate the quality of the ice and to refine the data collection strategy. Out of the 766 micrographs collected in total, 564 micrographs were retained after discarding the bad micrographs. Dose-weighted motion correction was done on the movie frames using MotionCor2^80^, and the CTF was estimated using CTFFIND4^81^. The rest of the processing was done in RELION 3.0^82^ using GPU acceleration wherever possible. 348,898 particles were picked with the RELION auto-picker and sorted by 2D and 3D classification, as well as by Z-score. The best particles were then selected for 3D refinement. Reference free 2D classification was performed iteratively to remove particle images containing ice contamination or bad particles. Approximately 36% of the initial number of particles were retained for further analysis by 3D classification. Both 3D classification and 3D refinement were done without applying any symmetric constraints (Table S1). The NCP structure (EMD-8140^25^) that was low pass filtered to 80 Å was used as a reference model for the initial round of 3D classification. Iterative rounds of 3D-classification led to the selection of the most homogenous particles that gave the final chromatosome structure. At the end of the 2D and 3D classification procedures, 46,196 particle images were used to obtain the refined 177-Chromatosome structure. After the 3D auto-refinement, the particles were polished, refined again followed by map sharpening and masking.

The resolution was estimated using Fourier Shell Correlation at gold standard, FSC = 0.143, cutoff. The B-factor was calculated automatically in RELION 3.0 for sharpening the final 177 bp chromatosome structure. The local resolution of the map was calculated in RELION and visualised in Chimera^83,84^. The Cryo-EM map was visualised using UCSF Chimera^83^. The hH1.0 structure (PDB ID: 6N89, chain B) was used as a template to generate the homology model of the linker histone in I-TASSER^85^. hH1.0 generated from homology modelling, and the NCP-601L model (PDB ID: 3LZ0) were rigid-body fitted into the EM density in UCSF Chimera^83^. The linker DNA was modelled in COOT^86^ and structure was regularised using geometry minimisation in PHENIX^87^.

### Precipitation assay

Nucleosome or chromatosome solutions (10 µl in 10 mM Tris–HCl, pH 7.5) with OD_260_ = 2 were mixed with 10 µl of MgCl_2_ or CoHexCl_3_ salts in the same buffer. Twenty to thirty mixtures with different Mg^2+^ or CoHex^3+^ concentrations were incubated 15 min at room temperature, followed by centrifugation at 20,000*g* for 15 min. OD_260_ of the supernatant was measured and plotted against cation concentration. Three runs of PA titrations were performed independently (Fig. 5 and Fig. S5). The plot was fitted by Boltzmann sigmoidal function using Origin Pro software (Origin Lab, Northampton, MA) and the midpoint of the fitted curve was set as EC_50_ value, which denotes the salt concentration of 50% sample precipitation.

### Small-angle X-ray scattering (SAXS)

Nucleosomes and chromatosomes were concentrated using Amicon ultra centrifugal filter (MWCO 10 kDa) (Merck). For studies of the 177 bp nucleosome and chromatosome in solution, the samples were prepared at 3 mg/ml concentration in 10 mM KCl and 10 mM Tris (pH 7.5). For the study of the aggregated nucleosome and chromatosome, the samples were prepared at 8 mg/ml concentration and precipitated by different concentrations of Mg^2+^. Precipitated samples were packed into quartz capillary tubes (1.5 mm diameter) and sealed with wax. SAXS data collection was performed at the beamline 23A workstation in the National Synchrotron Radiation Research Center (NSRRC), Taiwan^88^. The SAXS solution data were analysed by Primus software^89^ from the ATSAS package (version 2.8.4)^90,91^.

Reference molecular structures used for calculation of simulated SAXS form factors were generated from the published crystal structure of the 197 bp chromatosome^30^ and from the NCP structure generated in all-atom molecular dynamics (MD) simulations using 1KX5.pdb^92^ as a starting model. The MD-generated structure differs from the initial crystal structure by having basic histone tails collapsed on the negatively charged DNA, thus closely resembling the NCP in solution. Molecular models for the 177 bp nucleosome were obtained by attachment of the straight B-form DNA with length and sequence matching the corresponding experimentally studied NCPs. The structures of straight DNAs were generated by the NAB program^93^. To model unwrapping of the DNA from the histone core, selected numbers of the DNA base pairs in the 177 bp nucleosome structure were replaced by stretches of straight B-form DNA of the same sequence from one or both ends of the NCP. Explicit hydrogen atoms were added to the crystal structures. Chimera molecular manipulation and visualisation package was used for the generation of the structures^94^. CRYSOL module of the ATSAS package was used to fit experimental SAXS profiles to the form factors of molecular structures. The range of fitting was between 0.05 and 0.25 Å^−1^ to eliminate the effects of particle interaction in the low range of q and experimental noise at high q values. The radii of gyration (R_g_) of the 177 bp nucleosome and the chromatosome were calculated from the distance distribution function P(r) and the slope of the Guinier plot of the SAXS spectra, using respective modules of the ATSAS software^90,91^.

## Supplementary Information

Supplementary Information.

## Data Availability

The accession code for the deposited Cryo-EM map of the 177 bp chromatosome reported in this paper is EMD-30161 (Figs. 3, 4), and the PDB code for the homology model fitted hH1.0 is 7DBP.

## Abbreviations

aa: Amino acid
Cryo-EM: Cryogenic electron microscopy
CTD: C-terminal domain
NTD: N-terminal domain
GH: H1 globular domain
HO: Histone octamer
hHO: Human histone octamer
MD: Molecular dynamics
NCP: Nucleosome core particle
OD: Optical density
PA: Precipitation assay
SAXS: Small-angle X-ray scattering
