# Supplementary material for: Linker histone defines structure and self-association behaviour of the 177 bp human chromatosome
Source: Sci Rep. 2021 Jan 11;11:380. doi: 10.1038/s41598-020-79654-8 (PMC7801413; doi:10.1038/s41598-020-79654-8)
Supplement: Supplementary file 1 — Supplementary Information. [file 41598_2020_79654_MOESM1_ESM.pdf]

Supplementary data for the paper

Linker Histone Defines Structure and Self-Association Behaviour of the 177 bp Human Chromatosome

Sai Wang, Vinod K. Vogirala, Aghil Soman, Nikolay V. Berezhnoy, Zhehui Barry Liu, Andrew S.W. Wong, Nikolay Korolev, Chun-Jen Su, Sara Sandin and Lars Nordenskiöld

Figure S1.

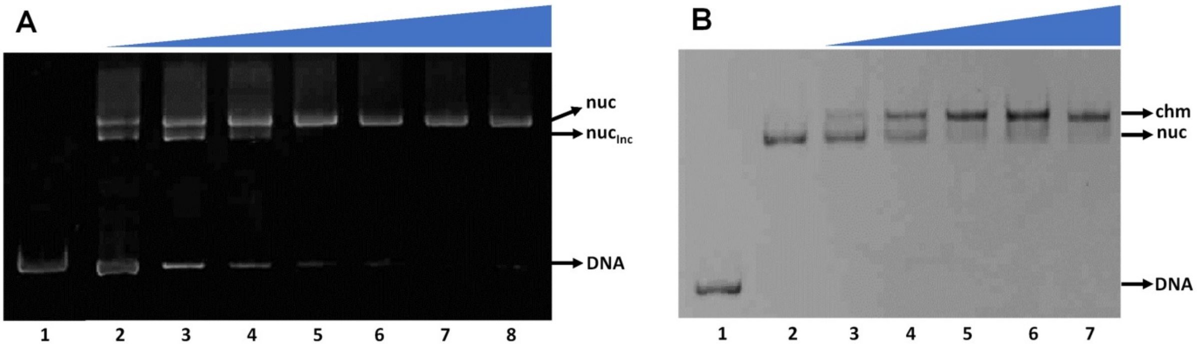

Figure S1. (**A**) EMSA analysis of the 177 bp-nucleosome samples on 6 % native PAGE. From left to right, lane 1 is 177 bp DNA as a marker; lanes 2 to 8 are the reconstituted 177 bp nucleosome samples with increasing HO to 177 bp DNA molar ratios from 0.4:1 to 1.4:1 (0.4, 0.8, 0.9, 1.0, 1.1, 1.2, 1.4, respectively). From top to bottom, as labelled to the right of the gel picture, "nuc", 177 bp nucleosome composed of one molecule of 177 bp DNA and one HO; "nuc_{inc}", incomplete nucleosome composed of the 177 bp DNA and incomplete HO (histone hexamer or tetramer); "DNA", 177 bp DNA. (**B**) EMSA analysis of the 177 bp chromatosome samples on 5 % native PAGE. From left to right, lane 1 and 2 are respectively the 177 bp DNA and 177 bp nucleosome loaded as markers; lanes 3 to 7 are the reconstituted 177 bp chromatosome samples with increasing $H1^0$ to 177 bp DNA molar ratios from 0.4:1 to 1.5:1 (0.4, 0.8, 1.2, 1.4, 1.5, respectively). From top to bottom, as labelled to the right of the gel picture, "chm", 177 bp chromatosome composed of one molecule of 177 bp DNA, one HO, and one molecule of linker histone hH1.0; "nuc", 177 bp nucleosome without bound linker histone; "DNA", 177 bp DNA.

Figure S2.

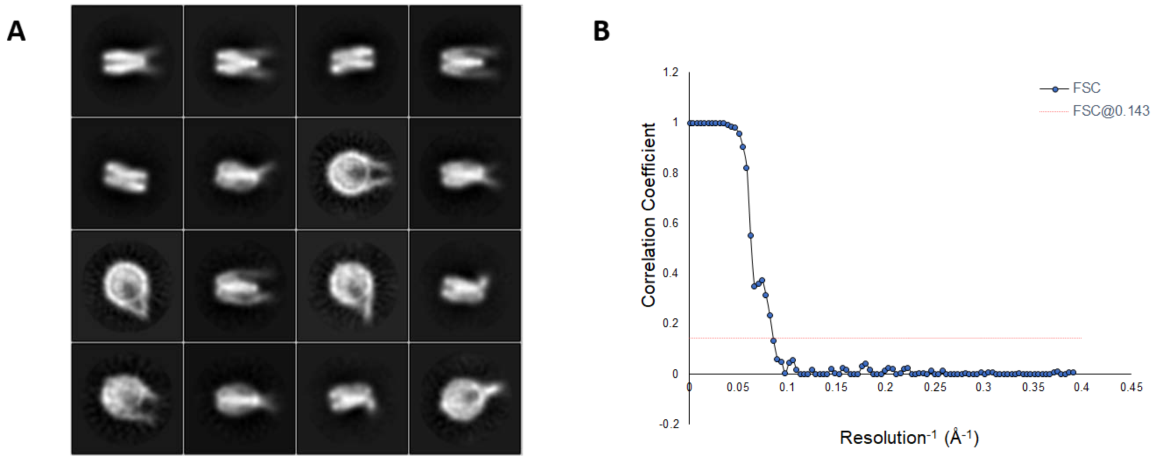

Figure S2. Cryo-EM single particle analysis of the 177 bp nucleosome. (**A**) 2D class averages. (**B**) Fourier shell correlation (FSC) plots for the EM density map. The resolution was estimated at 12 Å at FSC=0.143.

Figure S3.

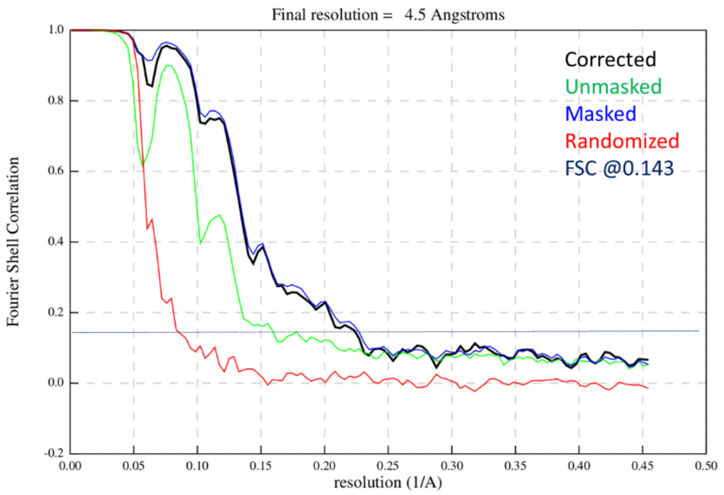

Figure S3. Fourier shell correlation (FSC) plots for the EM density map of the 177 bp chromatosome. The resolution was estimated at 4.5 Å at FSC=0.143.

Table S1. Comparison of the experimental SAXS spectra recorded for the 177 bp nucleosome and chromatosome to the form factors calculated for modelled molecular structures. Rows with numbers in bold shows parameters obtained from the experiment; section below presents the output of the analysis of the modelled molecules using published crystal structures [1, 2] and MD simulations; best fit χ^2 and D_{max} values highlighted by bold font. The fitting data obtained for the molecular models with the significant unwinding of the DNA from the histone core produces poor fitting (high χ^2 values) and is not shown.

Sample	R_g Guinier, Å	R_g from P(r), Å	D_{max}, Å	Quality*, χ^2
177 bp nucleosome, experimental SAXS spectrum				
Solution 3.0 mg/mL	**46.51** +/– 0.65	**47.58** +/– 0.65	**155.8**	0.938, excellent
Comparison with atomic structures				
177 bp nucleosome from 4nl0.pdb	46.35	46.64	**156.4**	13.59
Generated from MD data, 0-0**	47.21	47.28	163.2	**5.83**
Generated from MD data, 10-0**	48.03	48.08	163.7	15.07
177 bp chromatosome from 4nl0.pdb§	47.08	47.18	156.7	8.30
177 bp chromatosome, experimental SAXS spectrum				
Solution 3.0 mg/mL	**48.81**+/– 0.42	**49.43** +/– 0.40	**168.0**	0.814, good
Comparison with atomic structures				
177 bp chromatosome from 4nl0.pdb	47.08	47.18	156.7	**5.72**
177 bp nucleosome from 4nl0.pdb§	46.35	46.64	156.4	19.88
Generated from MD data, 0-0**,§	47.21	47.28	163.2	**2.57**
Generated from MD data, 10-0**,§	48.03	48.08	163.7	**3.17**
Generated from MD data, 15-0**,§	49.33	49.36	173.7	9.50
Generated from MD data, 10-10**,§	47.58	47.68	**163.7**	7.35

* For the experimental data, fit quality is given by number calculated using the GNOM module of the ATSAS package [3, 4]. For the experiment versus simulated fittings, the fitting quality is defined by χ^2 value calculated by CRYSOL module of the same package.

** Atomic structures of the 177 bp nucleosome were constructed from a snapshot of all-atom MD simulation of the 147 bp nucleosome and stretches of straight B-form DNA structures. Numbers indicate how many DNA base pairs are unwound from the histone core.

§ For this structure, comparison to the experimental spectrum was carried out as negative control.

Figure S4.

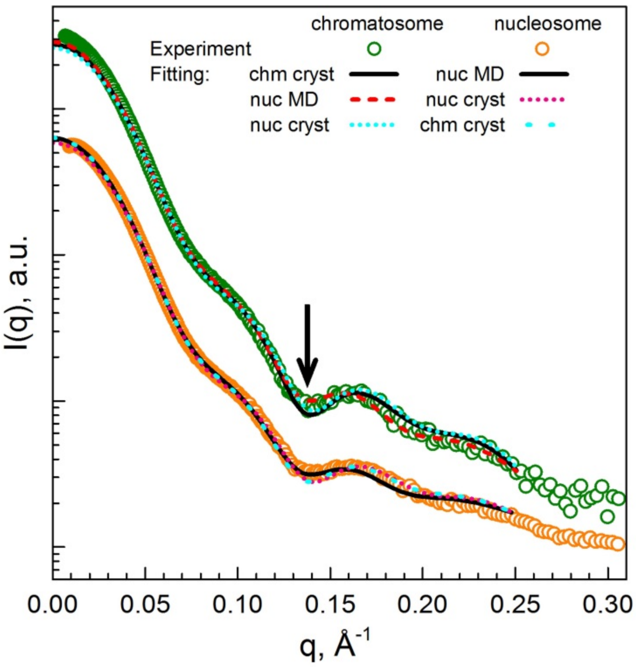

Figure S4. Comparison of SAXS spectra measured in solutions of the 177 bp nucleosome (orange symbols) and chromatosome (green symbols) to the SAXS form factors calculated for the molecular structures modelling 177 bp nucleosome and chromatosome (lines). SAXS spectra were recorded at 3 mg/ml concentration of the nucleosome or chromatosome in 10 mM KCl and 10 mM Tris (pH 7.5). Molecular structures for calculation of the SAXS form factors were built using a snapshot from the all-atom MD simulations of the 147 bp NCP or from the published crystal structure of the 197 bp chromatosome 5nl0.pdb, see materials and Methods section for details. Abbreviation used in the legend: “chm cryst”, 177 bp chromatosome obtained from the 5nl0.pdb structure; “nuc MD”, 177 bp nucleosome created from the coordinates of the MD snapshot and 20 bp straight linker DNA in ideal B-form; “nuc cryst”, 177 bp nucleosome generated by removing coordinates of the linker histone from the 177 bp chromatosome based on the 5nl0.pdb structure. See Table S1 above for a summary of the fitting results. An arrow indicates the signature dip at q=0.14 Å^{-1}.

Figure S5.

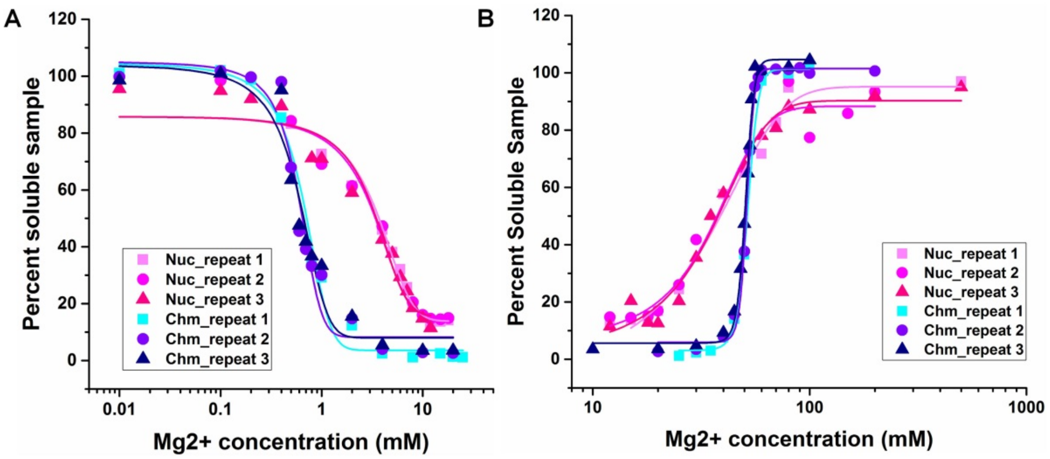

Figure S5. Analysis of the Mg^{2+}-induced precipitation (**A**) and resolubilisation (**B**) of the 177 bp nucleosome and chromatosome. Three independent repeats were carried out for each PA experiment. Points (square, circle or triangle) are the experimental data, solid lines are the results of fitting using Boltzmann sigmoidal function. In the legends, data for the nucleosome and chromatosome are marked respectively as “Nuc” and “Chm”.

Table S2. EC_{50} of Mg^{2+} induced 177 bp nucleosome and chromatosome precipitation and resolubilisation.

	Precipitation EC_{50} (mM)	Resolubilisation EC_{50} (mM)
177 bp nucleosome	3.0 +/− 0.1	34.2 +/− 0.3
177 bp chromatosome	0.6 +/− 0.0	51.2 +/− 0.6

Figure S6.

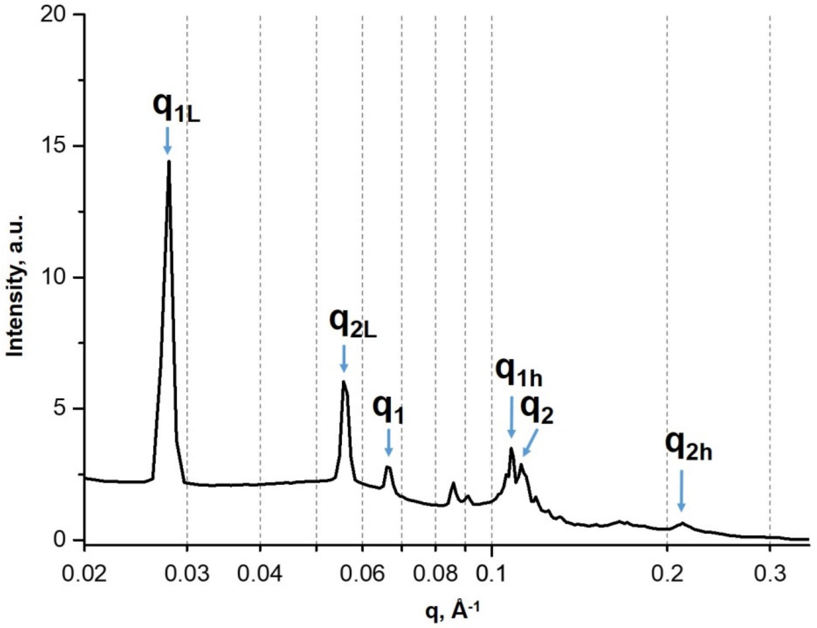

Figure S6. SAXS spectrum of chromatosome aggregated by 30 mM Mg^{2+}. Diffraction peaks indicating chromatosome stacking (q_{1h} and q_{2h}), a hexagonal arrangement of columns (q_1 and q_2), and lamello-columnar phase (q_{1L} and q_{2L}) are indicated. The appearance of this spectrum is different from the one in Figure 7 of the main text because the logarithmic scattering vector and linear intensity axes are used.

Supporting references

[1] C.A. Davey, D.F. Sargent, K. Luger, A.W. Maeder, T.J. Richmond, Solvent mediated interactions in the structure of nucleosome core particle at 1.9 Å resolution, J. Mol. Biol. 319 (2002) 1097-1113.

[2] J. Bednar, I. Garcia-Saez, R. Boopathi, A.R. Cutter, G. Papai, A. Reymer, et al., Structure and dynamics of a 197 bp nucleosome in complex with linker histone H1, Mol. Cell 66 (2017) 384-397.e388.

[3] D. Franke, M.V. Petoukhov, P.V. Konarev, A. Panjkovich, A. Tuukkanen, H.D.T. Mertens, et al., ATSAS 2.8: a comprehensive data analysis suite for small-angle scattering from macromolecular solutions, J. Appl. Crystallogr. 50 (2017) 1212-1225.

[4] M.V. Petoukhov, D. Franke, A.V. Shkumatov, G. Tria, A.G. Kikhney, M. Gajda, et al., New developments in the ATSAS program package for small-angle scattering data analysis, J. Appl. Crystallogr. 45 (2012) 342-350.
